# Sodium Butyrate Mitigates *Pseudomonas aeruginosa* Infection in bMECs Associated with the Modulation of TLR4/MAPK Pathway and Improvement of Autophagic Markers

**DOI:** 10.3390/vetsci13090925

**Published:** 2026-09-08

**Authors:** Xiaoli Shi, Yi Xu, Abdulrahman S. Alharthi, Tianle Xu

**Affiliations:** 1 College of Animal Science, Nanjing Agricultural University, Nanjing 210095, China; shixiaoli@njau.edu.cn; 2 College of Veterinary Medicine, Yangzhou University, Yangzhou 225009, China; 3 Department of Animal Production, College of Food and Agriculture Science, King Saud University, Riyadh 11451, Saudi Arabia; 4 Joint International Research Laboratory of Agriculture and Agri-Product Safety, Ministry of Education of China, Yangzhou University, Yangzhou 225009, China

**Keywords:** bovine mastitis, *Pseudomonas aeruginosa*, sodium butyrate, autophagic flux, TLR4/MAPK pathway, transcriptomics

## Abstract

*Pseudomonas aeruginosa* (PA) is a formidable environmental pathogen. It causes severe and refractory bovine mastitis. The escalating threat of antimicrobial resistance requires new non-antibiotic therapies. These alternative therapies should focus on targeting host-directed responses. Sodium butyrate (SB) is a prominent short-chain fatty acid. It possesses potent immunomodulatory properties. However, its protective mechanisms against PA-induced mammary injury remain elusive. This study investigated the efficacy and underlying molecular mechanisms of SB. bMECs were pretreated with 0.5 mmol/L SB for 18 h prior to challenge with *P. aeruginosa* (1 × 10^7^ CFU/mL, 6 h). We evaluated its ability to alleviate PA-induced cytotoxicity in bovine mammary epithelial cells (bMECs). Flow cytometry and ELISA demonstrated the strong protective effects of SB. SB pretreatment significantly reduced PA-induced cellular apoptosis. It also suppressed the hypersecretion of pro-inflammatory cytokines, including IL-6 and TNF-α. Next, transcriptomic sequencing (RNA-seq) was performed. We identified 589 differentially expressed genes (DEGs) between the PA-challenged and SB-treated groups. These DEGs were significantly enriched in the Toll-like receptor (*Tlr*), *Mapk*, and autophagy signaling pathways. We subsequently conducted molecular validations via RT-qPCR, Western blotting, and immunofluorescence. The results revealed that SB significantly suppressed the overactivation of the TLR4/MAPK cascade. Specifically, SB significantly downregulated the expression of TLR4. It also decreased the downstream phosphorylation levels of p38, ERK, and JNK. Furthermore, PA infection induced a severe blockade of autophagic flux. This dysfunction was evidenced by the concurrent cellular accumulation of LC3-II and the autophagic substrate p62. Remarkably, SB intervention was associated with the reduction in autophagic marker accumulation, evidenced by facilitated lysosomal clearance of p62. Collectively, sodium butyrate protects bMECs against PA-induced inflammation and apoptosis. These protective effects are closely associated with the suppression of the TLR4/MAPK signaling cascade and the alleviation of autophagic marker accumulation. This highlights the potential of SB as a promising preventive strategy for the clinical management of bovine mastitis.

## Simple Summary

Bovine mastitis caused by *Pseudomonas aeruginosa* is a severe challenge for the global dairy industry, and the rising resistance of this pathogen to antibiotics makes new, non-antibiotic strategies urgently needed. This study explored sodium butyrate, a naturally occurring compound with well-known immune-regulating properties, as a potential non-antibiotic treatment. We found that the bacteria damage cow udder cells by triggering massive inflammation and disrupting their natural autophagy. Remarkably, treating the cells with sodium butyrate successfully protected them by dampening the inflammatory process and restoring their normal cellular cleaning process. These findings highlight sodium butyrate as a promising non-antibiotic immunomodulatory candidate that warrants further in vivo validation for the prevention of bovine mastitis.

## 1. Introduction

Bovine mastitis remains one of the most economically devastating diseases in the global dairy industry, leading to compromised animal welfare, reduced milk yield, and significant financial losses [1,2]. Among the diverse array of mastitis-causing pathogens, *Pseudomonas aeruginosa* (PA) has increasingly emerged as a formidable environmental opportunistic pathogen [3]. PA infections in the bovine mammary gland frequently result in acute or peracute clinical mastitis, which is notoriously difficult to eradicate due to the pathogen’s intrinsic resistance to multiple antibiotics and its repertoire of virulence factors [4]. The escalating crisis of antimicrobial resistance necessitates the urgent exploration of novel, host-directed therapeutic strategies that focus on modulating the local immune microenvironment rather than solely targeting the pathogen [5].

Bovine mammary epithelial cells (bMECs) act not only as a crucial physical barrier but also as the primary sentinels of the innate immune system within the udder [6]. Upon PA invasion, bMECs initiate inflammatory responses characterized by the massive secretion of pro-inflammatory cytokines, including TNF-α, IL-6, and IL-1β [7]. This inflammatory cascade is primarily orchestrated through pattern recognition receptors, notably Toll-like receptor 4 (TLR4), which recognizes pathogen-associated molecular patterns and subsequently activates downstream signaling networks, such as the mitogen-activated protein kinase (MAPK) pathway [8,9]. Furthermore, emerging evidence highlights that PA infection profoundly disrupts cellular homeostasis by inducing apoptosis and dysregulating autophagy [10,11]. Autophagy, a highly conserved catabolic process, is critical for clearing intracellular pathogens and maintaining homeostasis [12]; however, severe pathogen-induced stress can lead to autophagic dysfunction, resulting in the aberrant accumulation of autophagosomes, exacerbated inflammatory signaling, and accelerated cellular damage [13,14]. The intricate crosstalk between the TLR4/MAPK inflammatory axis and autophagic flux during PA infection in bMECs remains a critical gap in our understanding of mastitis pathogenesis.

In recent years, short-chain fatty acids (SCFAs) have garnered significant attention as potent modulators of host metabolic and immune responses [15,16,17]. Sodium butyrate (SB), a representative SCFA and well-known histone deacetylase (HDAC) inhibitor, has demonstrated remarkable anti-inflammatory, barrier-protecting, and stress-alleviating properties in various gastrointestinal and systemic disease models [18]. Despite its proven efficacy in mitigating cellular stress and restoring homeostasis, the therapeutic potential of SB against PA-induced mastitis—and specifically, its capacity to regulate the TLR4/MAPK signaling cascade and restore autophagic flux in bMECs—is largely unexplored [19,20].

Therefore, this study aimed to elucidate the precise molecular mechanisms underlying PA-induced inflammation, apoptosis, and autophagic alterations in bMECs, and to systematically evaluate the protective efficacy of SB. By integrating transcriptomic sequencing (RNA-seq) with in vitro molecular validation, we sought to delineate how SB interventions may reprogram the cellular response to PA infection, specifically targeting the TLR4/MAPK pathway and autophagy mechanisms. Our findings are expected to bridge the existing knowledge gap and provide robust theoretical foundations for utilizing SB as a promising, non-antibiotic strategy for the clinical management of PA-associated bovine mastitis.

## 2. Materials and Methods

### 2.1. Cell Culture and Experimental Design

Culture of primary bMECs was performed as described previously [21]. Bovine mammary tissue obtained from three mid-lactating Holstein cows (30.26 ± 3.1 kg/d of milk yield, 2.67 parity, and 175 ± 6 DIM) without incidence of clinical disease was used for cell isolation and purification. Cells from each cow were pooled into a single sample. Cells were cryopreserved in liquid nitrogen and recovered following standard procedures prior to experimental use. All experiments were performed with cells at the fourth to sixth passage. Bovine mammary epithelial cells (bMECs) were cultured and maintained in growth medium supplemented with 10% (*v*/*v*) fetal bovine serum (FBS) under standard physiological conditions (37 °C, 5% CO_2_). To investigate the protective mechanisms of sodium butyrate (SB) against *Pseudomonas aeruginosa* (PA)-induced inflammatory and autophagic damage, bMECs were randomly divided into four experimental groups: the control group (**Ctrl**), the sodium butyrate alone group (**SB**), the *P. aeruginosa* infection group (**PA**), and the sodium butyrate treatment group (**SP**). Specifically, the Ctrl group was cultured in an equivalent volume of PBS or standard vehicle. The SB group was treated with optimized concentrations of SB alone (0.5 mmol/L). For the PA group, bMECs were challenged with *P. aeruginosa* (1 × 10^7^ CFU/mL) for 6 h to establish the in vitro mastitis model. For the SP treatment group, bMECs were pre-treated with SB for 18 h and subsequently challenged with PA for 6 h. Cells were confirmed to be free of mycoplasma contamination prior to use. For the bacterial challenge, the standard *P. aeruginosa* strain PAO1 was cultured in Luria–Bertani (LB) broth at 37 °C to the mid-logarithmic phase. The bacteria were pelleted, washed, and resuspended in PBS to achieve the desired Multiplicity of Infection (MOI) of approximately 50:1 (equivalent to 1 × 10^7^ CFU/mL). The precise optimal concentrations of PA and SB, as well as the optimal temporal dynamics for the inflammatory responses, were determined based on preliminary dose-dependence assays and previously established protocols [22].

### 2.2. Electron Microscopy Analysis (SEM and TEM)

To evaluate cell surface morphology and intracellular ultrastructural alterations (particularly mitochondrial integrity and autophagosome formation), scanning electron microscopy (SEM) and transmission electron microscopy (TEM) were employed. For **SEM**, bMECs grown on coverslips were fixed with 2.5% glutaraldehyde at 4 °C overnight. Following three washes with PBS, the samples were post-fixed with 1% osmium tetroxide, dehydrated through a graded ethanol series (50%, 70%, 80%, 90%, and 100%), and subjected to critical point drying. The specimens were then sputter-coated with gold and observed under a scanning electron microscope to assess cytomorphology and microvilli integrity. For **TEM**, bMECs were collected via centrifugation and fixed in 2.5% glutaraldehyde, followed by post-fixation in 1% osmium tetroxide. The cell pellets were dehydrated in a graded series of ethanol and acetone, and subsequently embedded in epoxy resin. Ultrathin sections (70–90 nm) were cut using an ultramicrotome, stained with uranyl acetate and lead citrate, and examined under a transmission electron microscope. The autophagic structures (autophagosomes and autolysosomes) and morphological changes in mitochondria were analyzed.

### 2.3. Flow Cytometry for Apoptosis Detection

The apoptotic effect of PA infection on bMECs and the potential protective role of SB were evaluated using an Annexin V/PI apoptosis detection kit (Sigma-Aldrich, Burlington, MA, USA). Briefly, bMECs were seeded into 6-well plates at a density of 2 × 10^5^ cells/well and subjected to the designated treatments (Ctrl, SB, PA, SP). Post-treatment, cells were harvested via gentle trypsinization, washed with cold PBS, and resuspended in binding buffer. The cells were then double-stained with Annexin V-FITC and Propidium Iodide (PI) in the dark according to the manufacturer’s instructions. The percentage of apoptotic cells was quantitatively analyzed utilizing a FACSCalibur flow cytometer platform (BD Biosciences, Franklin Lakes, NJ, USA).

### 2.4. ELISA for Cytokine Production

The secretion levels of pro-inflammatory cytokines in the bMEC culture medium were quantified by Enzyme-Linked Immunosorbent Assay (ELISA). Following the respective treatments, cytokine concentrations were measured directly from the culture supernatants of equally seeded cells. The concentrations of Interleukin-6 (IL-6) and Tumor Necrosis Factor-alpha (TNF-α) were measured using the DuoSet ELISA bovine IL-6 (DY8190) and DuoSet ELISA bovine TNF-α kits (DY2279) (R&D Systems, Minneapolis, MN, USA), strictly following the manufacturer’s protocols. The evaluation reports for the sensitivity and specificity of these kits could be found at https://www.rndsystems.com/. The optical density was measured utilizing a microplate reader.

### 2.5. Transcriptomic Sequencing and Analysis

After RNA extraction from the treated bMECs (three independent biological replicates per group), RNA integrity and purity were assessed. We used the Agilent 2100 Bioanalyzer (Agilent Technologies, Santa Clara, CA, USA). Only samples with an RNA Integrity Number (RIN) ≥ 7.0 were submitted for transcriptomic sequencing. Sequencing was performed by Majorbio Biotechnology Co., Ltd. (Shanghai, China) on an Illumina HiSeq platform (2 × 150 bp read length). The raw sequencing data were subjected to strict quality control, yielding high-quality reads with Q20 > 98% and Q30 > 94%. The resulting clean reads were mapped to the Bos taurus reference genome (ARS-UCD1.2) using HISAT2 (version 2.1.0). Gene expression levels were quantified using RSEM (version 1.3.1). Differential expression analysis between the PA and SP groups was conducted using the DESeq2 R package (version 1.24.0). A standard two-group comparison design was applied. Principal Component Analysis (PCA) and functional enrichment analyses (GO and KEGG) were systematically performed utilizing the Majorbio Cloud Platform.

### 2.6. RNA Isolation and Real-Time Quantitative PCR

Total RNA was isolated from bMECs using TRIzol reagent (Invitrogen, Carlsbad, CA, USA) according to the manufacturer’s instructions. Approximately 1.5 μg of total RNA was reverse transcribed into cDNA using the Superscript II kit (Invitrogen, Thermo Fisher Scientific, Waltham, MA, USA)). Real-time quantitative PCR (qPCR) was conducted on an ABI 7300 system (Applied Biosystems, Foster City, CA) using the SYBR Premix EX Taq kit (TaKaRa, Shiga, Japan). Gene-specific primers for target genes (*Tlr4*, *Mapk14*, *Il6*, *Il1b*, *Map1lc3b*, *Sqstm1*, *Becn1*, and *Atg5*) were designed utilizing Premier 6.0 software, and their amplification efficiencies were validated prior to use (Supplementary Table S1). The geometric mean of three validated reference genes (GAPDH, UXT, RPS9) was used to ensure normalization stability. The relative mRNA expression levels were calculated based on the 2^−ΔΔCt^ method.

### 2.7. Western Blotting Analysis

Total cellular proteins were extracted from bMECs using RIPA lysis buffer (Beyotime, Shanghai, China) supplemented with protease and phosphatase inhibitor cocktails. Protein concentrations were quantified using a BCA protein assay kit (Beyotime Biotechnology, Shanghai, China). Equal amounts of protein extracts were separated by SDS-PAGE on 4% to 20% polyacrylamide gels and subsequently electrophoretically transferred onto nitrocellulose membranes (Millipore, Billerica, MA, USA). After blocking with 5% BSA, the membranes were incubated overnight at 4 °C with specific primary antibodies. All antibodies were purchased from Beyotime Biotechnology (Shanghai, China) and diluted at a ratio of 1:1000. The primary antibodies included: Rabbit anti-TLR4 (AF7017), Rabbit anti-phospho-p38 (Cat# AF5887), Rabbit anti-p38 (AF7668), Rabbit anti-phospho-ERK1/2 (AF5818), Rabbit anti-ERK1/2 (AF1051), Rabbit anti-phospho-JNK (AF5860), Rabbit anti-JNK (AF6022), Rabbit anti-LC3B (AF5402), Rabbit anti-p62/SQSTM1 (AF5384), Rabbit anti-Beclin-1 (AF5128), and Mouse anti-β-actin (AF5001). Following thorough washing with TBST, the membranes were incubated with horseradish peroxidase (HRP)-conjugated secondary antibodies at room temperature for 1 h. The secondary antibodies included Goat anti-Rabbit IgG (A0208, 1:2000 dilution) and Goat anti-Mouse IgG (A0216, 1:2000 dilution). Band intensities were quantified using ImageJ software (1.51j8) with local background subtraction, and each target was normalized to its corresponding β-actin signal from at least three independent biological replicates.

### 2.8. Immunofluorescence Analysis

To observe the expression and precise subcellular localization of TLR4, bMECs were cultured on sterile glass coverslips in 12-well plates (2 × 10^4^ cells/well). Following designated treatments, cells were washed with PBS and fixed with 4% paraformaldehyde for 15 min at room temperature. Permeabilization was performed using 0.3% Triton X-100 for 15 min. The cells were then blocked with 5% BSA for 1 h at 37 °C to prevent non-specific binding. Subsequently, the specimens were incubated overnight at 4 °C with a specific primary antibody against TLR4. After three washes with PBS, the cells were probed with a fluorochrome-conjugated secondary antibody for 1 h in the dark at 37 °C. Nuclei were counterstained with DAPI (1 μg/mL; Sigma-Aldrich, Burlington, MA, USA) for 5 min. The fluorescent signals and target localization were visualized and captured using an LSM 710 confocal laser scanning microscope (Zeiss, Oberkochen, Germany).

### 2.9. Statistical Analysis

All quantitative data in this study are presented as the mean ± SEM. Unless otherwise noted, all assays were performed in triplicate, representing three independent in vitro culture and treatment experiments conducted on different days utilizing the established bMEC pool. Statistical analyses were predominantly performed utilizing GraphPad Prism software 11.0.2. Prior to the analysis, the data were tested for normal distribution using the Shapiro–Wilk test and for homogeneity of variance using Levene’s test. For comparisons among the four experimental groups (Ctrl, SB, PA, and SP), statistical significance was evaluated using a one-way analysis of variance (ANOVA), followed by Tukey’s multiple comparison post hoc test. For the transcriptomic (RNA-seq) data, differential expression analysis was conducted using the DESeq2 R package, and the resulting *p*-values were adjusted using the Benjamini and Hochberg’s approach for controlling the false discovery rate (FDR). Genes with an adjusted *p*-value (p-adj) < 0.05 and |log2(Fold Change)| > 1 were assigned as differentially expressed. Throughout the analyses, a value of *p* < 0.05 was considered to indicate a statistically significant difference, and *p* < 0.01 was considered highly significant.

## 3. Results

### 3.1. Sodium Butyrate Mitigates PA-Induced Apoptosis and Pro-Inflammatory Cytokine Hypersecretion in bMECs

**Figure 1:**
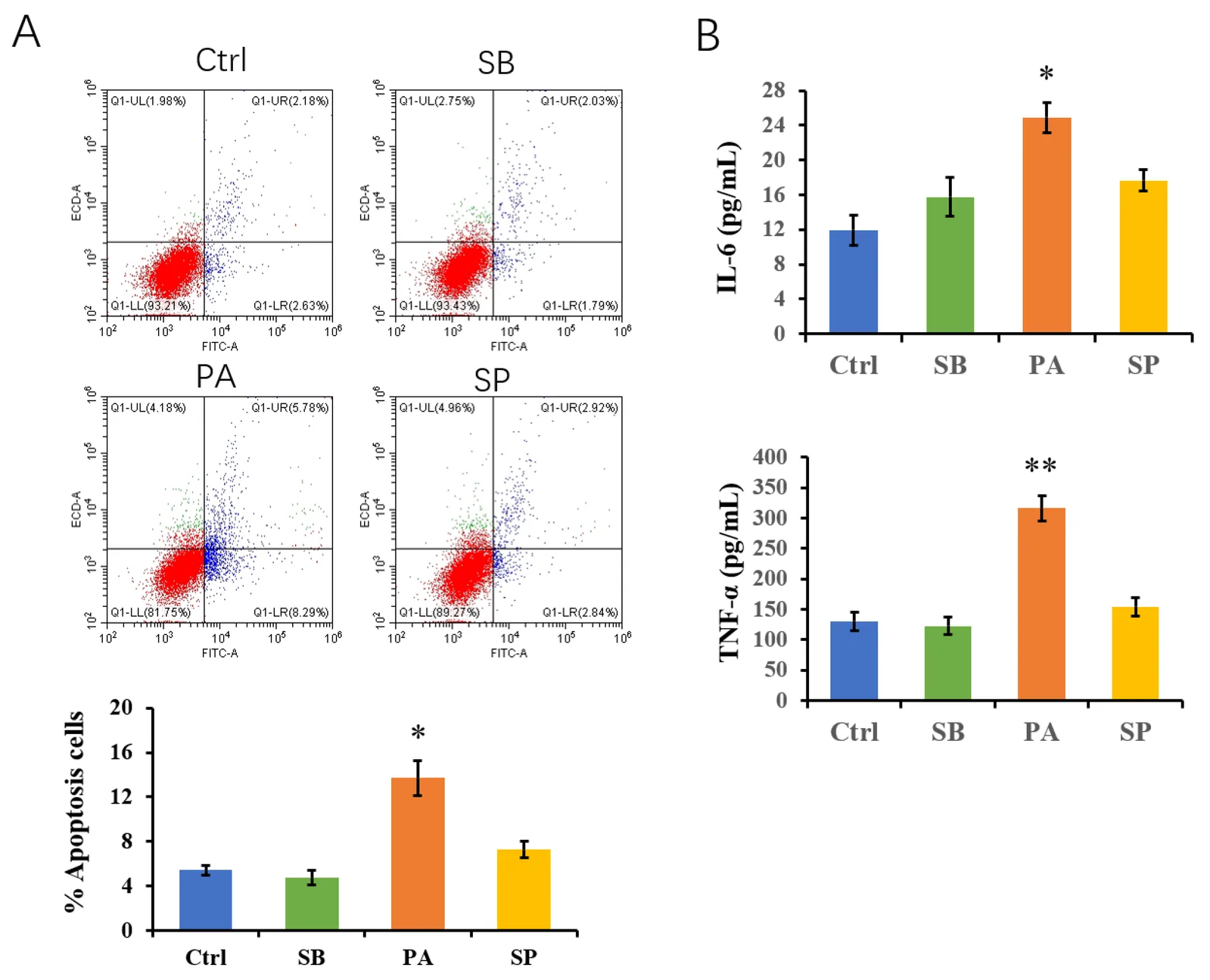
Sodium butyrate (SB) alleviates *Pseudomonas aeruginosa* (PA)-induced apoptosis and pro-inflammatory cytokine secretion in bovine mammary epithelial cells (bMECs). (**A**) The apoptosis of bMECs in the Ctrl, SB, PA, and SP groups was analyzed by flow cytometry using Annexin V-FITC/PI dual staining. The corresponding bar graph represents the quantitative statistical analysis of the total apoptotic cell percentage. (**B**) The concentrations of the pro-inflammatory cytokines IL-6 and TNF-α in the cell culture supernatants were measured by ELISA. Data are presented as the mean ± SEM from independent biological triplicates. * *p* < 0.05, ** *p* < 0.01.

To investigate the potential protective effects of sodium butyrate (SB) against *P. aeruginosa* (PA)-induced cytotoxicity, we first assessed the cellular apoptosis rate of bMECs using Annexin V-FITC/PI dual staining flow cytometry. As depicted in Figure 1A, the Ctrl and SB alone groups maintained a low basal level of apoptosis (approximately 6% and 5%, respectively). However, PA challenge significantly increased the proportion of apoptotic cells, particularly in the early (Q1-LR) and late (Q1-UR) apoptotic quadrants, resulting in a total apoptosis rate of 13.45 ± 1.2% (*p* < 0.05). Notably, pretreatment with SB in the SP group significantly blunted this effect, reducing the total apoptosis rate to 7.28 ± 0.77%.

To further evaluate the inflammatory response, the secretion levels of key pro-inflammatory cytokines in the culture supernatants were quantified using ELISA (Figure 1B). Following PA infection, the concentrations of IL-6 and TNF-α surged significantly (*p* < 0.05 and *p* < 0.01, respectively) compared to the uninfected Ctrl group, reaching approximately 26 pg/mL and 310 pg/mL. In contrast, SB intervention effectively suppressed this inflammatory storm, with IL-6 and TNF-α levels in the SP group sharply declining to roughly 16 pg/mL and 140 pg/mL. These initial findings confirm that SB alleviates PA-induced apoptosis and excessive inflammation without inducing cytotoxicity itself.

### 3.2. Transcriptomic Profiling Highlights the Modulation of Inflammatory and Autophagic Pathways by SB

**Figure 2:**
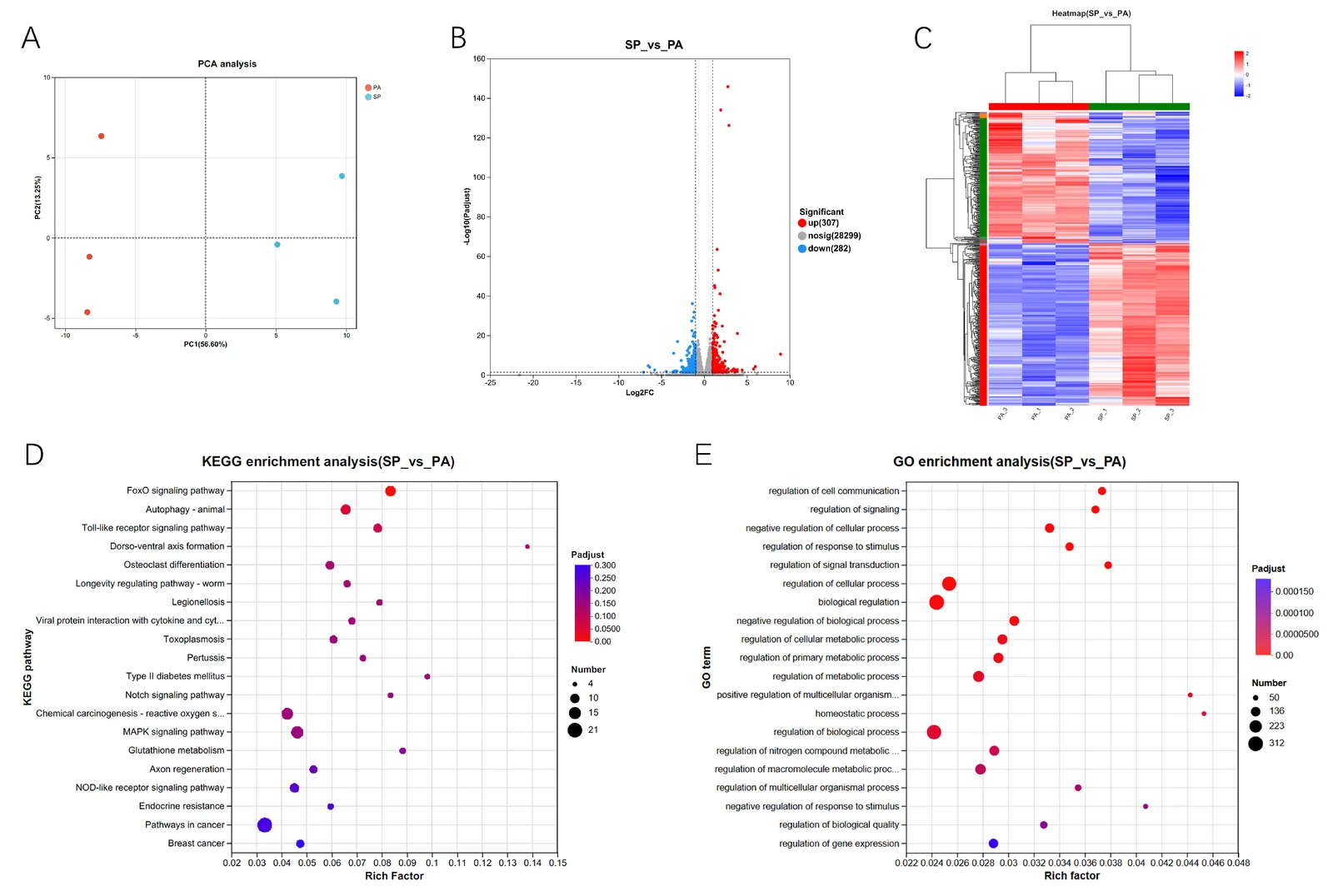
Transcriptomic analysis of differentially expressed genes (DEGs) between the PA-infected and SB-treated (SP) groups. (**A**) Principal component analysis (PCA) plot showing the spatial distribution and variance of the RNA-seq data from the PA and SP groups. (**B**) Volcano plot illustrating the DEGs. Red dots represent significantly upregulated genes, and blue dots represent significantly downregulated genes in the SP group compared to the PA group (|log2FoldChange| > 1 and p-adj < 0.05). (**C**) Hierarchical clustering heatmap displaying the distinct gene expression signatures of the DEGs. (**D**) Kyoto Encyclopedia of Genes and Genomes (KEGG) pathway enrichment analysis bubble chart of the DEGs. (**E**) Gene Ontology (GO) functional enrichment analysis bubble chart of the DEGs.

To comprehensively unravel the underlying molecular mechanisms of SB-mediated protection, RNA sequencing (RNA-seq) was performed to compare the transcriptomic profiles of the PA and SP groups. Principal component analysis (PCA) revealed a distinct spatial separation between the two groups (Figure 2A), with PC1 and PC2 accounting for 56.60% and 13.25% of the total variance, respectively, indicating robust reproducibility and significant transcriptomic shifts induced by SB.

Based on the criteria of |log2(Fold Change)| > 1 and *p*-adjust < 0.05, a total of 589 differentially expressed genes (DEGs) were identified via volcano plot (Figure 2B), comprising 307 upregulated and 282 downregulated genes in the SP group relative to the PA group. The hierarchical clustering heatmap further corroborated the distinct gene expression signatures between the two treatments (Figure 2C). Crucially, KEGG pathway enrichment analysis (Figure 2D) revealed that these DEGs were highly enriched in classical immune and homeostatic signaling cascades, notably the Toll-like receptor signaling pathway, MAPK signaling pathway, NOD-like receptor signaling pathway, and Autophagy-animal pathway. Consistent with this, Gene Ontology (GO) enrichment analysis (Figure 2E) indicated that the DEGs were predominantly involved in biological processes such as the regulation of cell communication, regulation of response to stimulus, and negative regulation of cellular processes.

### 3.3. SB Extensively Downregulates the Transcription of PA-Triggered Inflammatory and Autophagy-Related Genes

**Figure 3:**
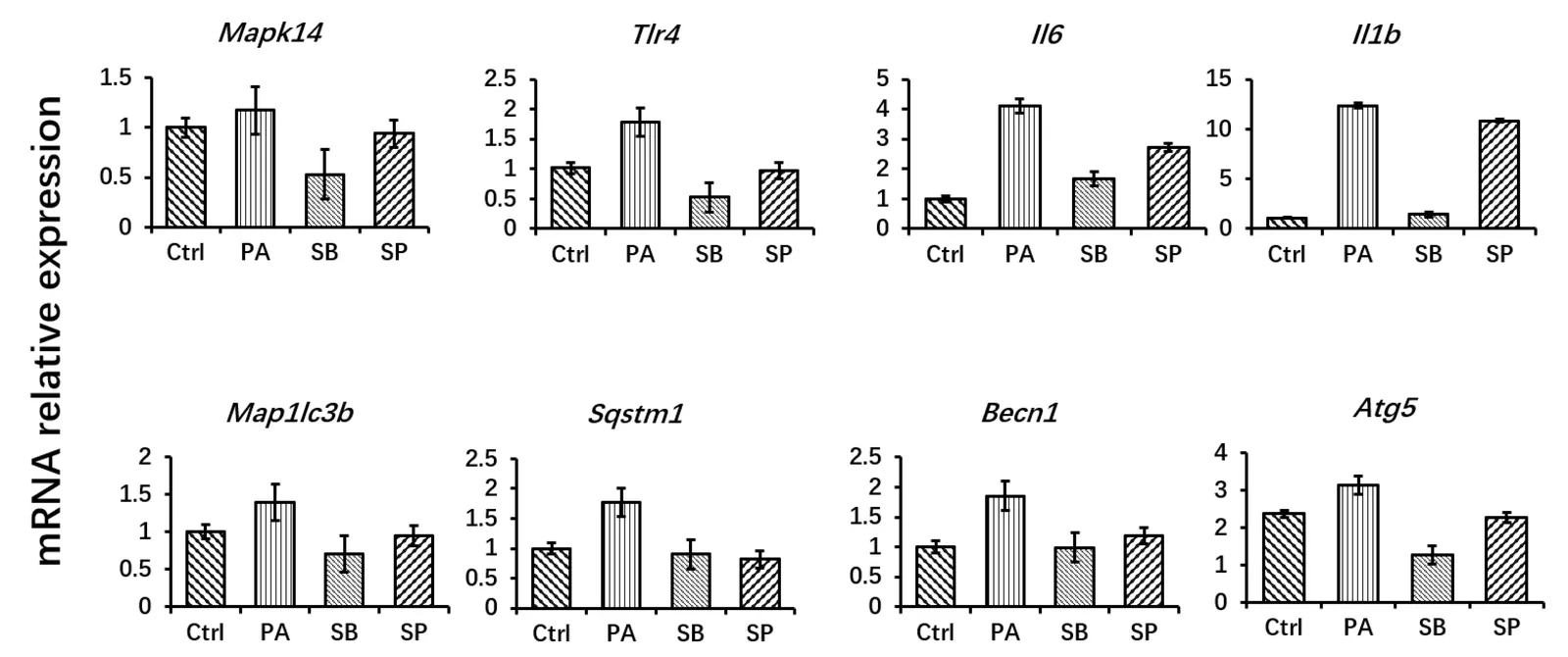
SB downregulates the transcription of inflammation- and autophagy-related genes in PA-infected bMECs. The relative mRNA expression levels of key inflammatory mediators and receptors (*Mapk14*, *Tlr4*, *Il6*, *Il1b*) and core autophagy-related genes (*Map1lc3b*, *Sqstm1*, *Becn1*, *Atg5*) were determined by RT-qPCR. The NC (Negative Control) group corresponds to the uninfected baseline. Internal reference genes were used for data normalization. Data are expressed as the mean ± SEM.

Guided by the transcriptomic enrichment results, we validated the mRNA expression levels of core genes associated with inflammation and autophagy using RT-qPCR (Figure 3). The results demonstrated that PA infection triggered a robust transcriptional response, significantly upregulating the mRNA levels of immune receptors and pro-inflammatory mediators, including *Tlr4*, *Mapk14* (p38), *Il6*, and *Il1b*, compared to the Ctrl group. Similarly, the transcription of core autophagy-related genes (*Map1lc3b*, *Sqstm1*, *Becn1*, and *Atg5*) was markedly elevated in the PA group, implying a strong autophagic initiation signal in response to the bacterial challenge. Remarkably, SB treatment in the SP group comprehensively reversed these trends, significantly downregulating the transcription of both the inflammatory and autophagic gene panels toward normal physiological levels.

### 3.4. SB Modulates PA-Induced Autophagic Protein Expression and Accumulation

**Figure 4:**
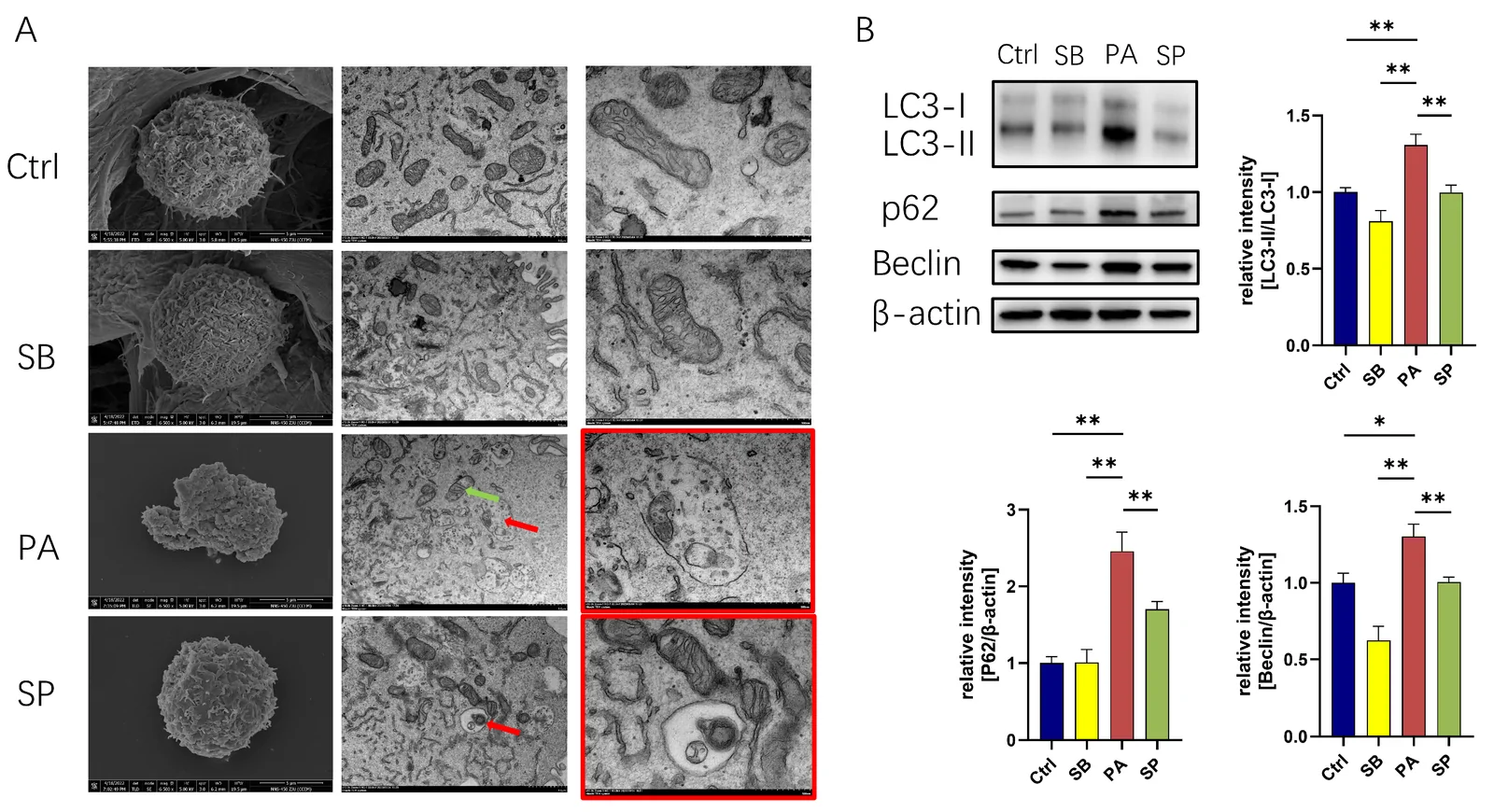
SB modulates PA-induced autophagic protein expression and modulates the expression of autophagic markers. (**A**) Representative Scanning Electron Microscopy (SEM; left, scale bar = [5 μm]) and Transmission Electron Microscopy (TEM; middle and right, scale bar = [1.0 μm and 500 nm]) images of bMECs. Red boxes indicate magnified TEM regions. Red arrows denote autophagic structures, and green arrows indicate damaged mitochondria. Cells were pretreated with 0.5 mmol/L SB for 18 h, followed by 1 × 10^7^ CFU/mL PA challenge for 6 h. (**B**) Western blot images and quantitative analysis of LC3, p62, and Beclin-1. β-actin was used as the loading control. Data are presented as the mean ± SEM (*n* = 3). * *p* < 0.05, ** *p* < 0.01 (the original Western blotting pictures can be found in Figure S1).

To ascertain whether the transcriptional alterations translated to functional changes in autophagic activity, the protein expression levels of key autophagic markers (LC3, p62, and Beclin-1) were examined via Western blotting (Figure 4A,B). Visual evaluation of the band intensities revealed that PA challenge induced a pronounced accumulation of LC3-II and Beclin-1, alongside a significant accumulation of the autophagic substrate p62, when compared to the Ctrl group. The concurrent buildup of LC3-II and p62 indicates that while PA initiates autophagosome formation, it severely impairs lysosomal degradation, leading to a blockade in the autophagic flux. Conversely, in the SP group, SB intervention visibly diminished the band densities of LC3-II and Beclin-1. More importantly, SB significantly promoted the clearance of p62 protein. The reduction in p62 accumulation strongly implies that SB not only modulates autophagic initiation but also alleviates the PA-induced p62 accumulation, suggesting a potential improvement in autophagic clearance.

### 3.5. SB Systematically Suppresses the Overactivation of the TLR4/MAPK Signaling Cascade

**Figure 5:**
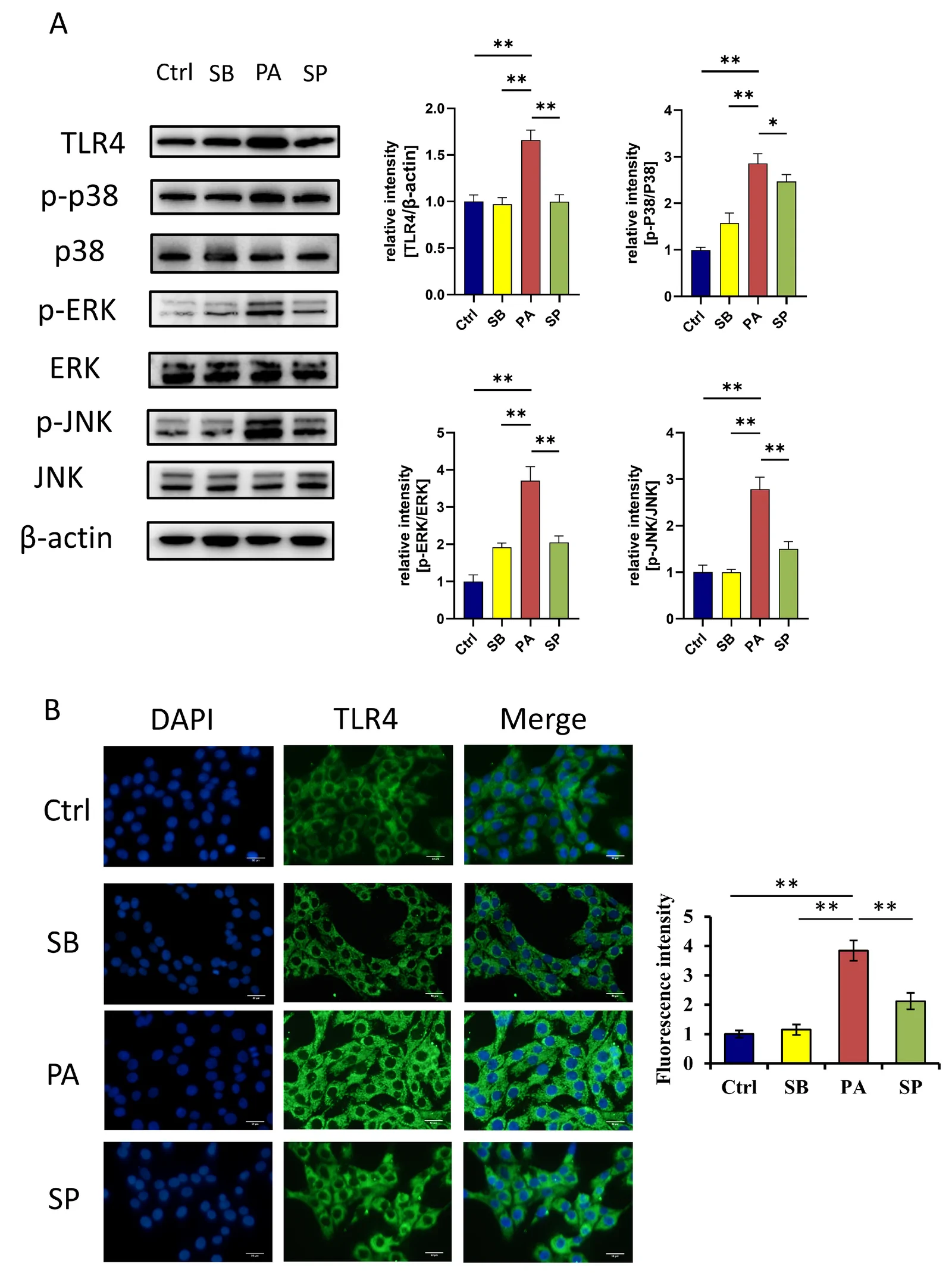
SB suppresses the overactivation of the TLR4/MAPK signaling cascade in PA-infected bMECs. (**A**) Representative Western blot images showing the total protein expression of TLR4 and the phosphorylation status of downstream MAPK cascade components, including p-p38/p38, p-ERK/ERK, and p-JNK/JNK. β-actin served as the internal loading control. (**B**) Representative immunofluorescence images illustrating the subcellular localization and fluorescence intensity of TLR4 (green) in bMECs across the different treatment groups. Cell nuclei were counterstained with DAPI (blue). Data are presented as the mean ± SEM (*n* = 3). * *p* < 0.05, ** *p* < 0.01 (the original Western blotting pictures can be found in Figure S1).

Given the critical role of TLR4 and MAPKs identified in our RNA-seq data, we meticulously evaluated their protein expression and phosphorylation status (Figure 5). Immunofluorescence staining (Figure 5B, top) provided clear in situ evidence: compared to the weak, diffuse signal in the Ctrl and SB groups, PA infection induced intense TLR4 expression (green fluorescence) heavily aggregated within the cytoplasm and membrane of bMECs. Following SB treatment, the fluorescence intensity of TLR4 in the SP group was visibly attenuated.

These morphological observations were highly consistent with the Western blot results (Figure 5A). The total protein expression of TLR4 was significantly upregulated by PA and subsequently suppressed by SB. Furthermore, analyzing the downstream MAPK cascade revealed that PA infection triggered the phosphorylation of p38, ERK, and JNK (manifested by dense p-p38, p-ERK, and p-JNK bands), without noticeably altering their respective total protein levels. In contrast, the SP group exhibited weaker bands for all three phosphorylated kinases compared to the PA group. Collectively, these data suggest that the cytoprotective and anti-inflammatory functions of SB are closely associated with the suppression of the TLR4/MAPK signaling axis.

## 4. Discussion

Bovine mastitis remains one of the most persistent and costly diseases affecting the global dairy industry [2]. While coliforms such as *Escherichia coli* have been extensively studied, *Pseudomonas aeruginosa* (PA) is increasingly recognized for causing acute, necrotic, and frequently refractory mastitis [4,7]. The virulence factors of PA, combined with the rising incidence of multidrug resistance, severely compromise traditional antibiotic therapies [3]. Consequently, identifying host-directed modulators capable of dampening infection-induced tissue damage is of profound clinical relevance. In the present study, we systematically demonstrated that sodium butyrate (SB), a prominent short-chain fatty acid (SCFA), effectively attenuates PA-induced cytotoxicity and apoptosis in bovine mammary epithelial cells (bMECs) by comprehensively suppressing the TLR4/MAPK inflammatory cascade and alleviating autophagic marker accumulation.

The mammary epithelium functions as the primary physical and immunological barrier against invading pathogens. Pathogen recognition is largely mediated by pattern recognition receptors, particularly Toll-like receptor 4 (TLR4) [23]. Upon PA challenge, bMECs rapidly mounted an immune response, evidenced by the marked upregulation of TLR4 and the downstream hyperphosphorylation of MAPKs, including p38, ERK, and JNK. While a basal inflammatory response is essential for pathogen clearance, the overactivation of the MAPK cascade drives the massive secretion of pro-inflammatory cytokines (IL-6, TNF-α, IL-1β) and accelerates cellular apoptosis, exacerbating tissue injury [24]. Our transcriptomic analysis and subsequent molecular validations revealed that SB intervention significantly repressed this inflammatory axis. As an endogenous SCFA and a well-characterized histone deacetylase (HDAC) inhibitor, SB has been widely documented for its potent immunomodulatory and barrier-protecting properties in various epithelial models [16,19,22]. Our results may extend these findings to the bovine mammary gland, indicating that SB serves as an effective brake against PA-induced hyperinflammation. This finding strongly aligns with recent studies demonstrating SB’s broad-spectrum immunomodulatory effects across various inflammatory models. For instance, recent evidence indicates that SB inhibits pathogen-induced inflammation in mammary epithelial cells via classical immune receptors [25], and exhibits remarkable efficacy in suppressing MAPKs or NF-κB activation in related inflammatory animal models [26,27]. By comparing these previous reports with our present findings, it becomes evident that SB’s protective capacity is versatile, extending its efficacy to Gram-negative opportunists like PA primarily by downregulating the TLR4/MAPK cascade.

Beyond classic inflammatory signaling, our study highlights the pivotal—and often paradoxical—role of autophagy during PA infection. Autophagy is a highly conserved intracellular degradation system essential for cellular homeostasis and defense [12]. During bacterial infections, functional autophagy targets intracellular pathogens for lysosomal degradation. However, severe oxidative stress and specific virulence factors from pathogens can hijack or stall this mechanism [13]. Our transmission electron microscopy (TEM) and Western blot analyses provided evidence of autophagic dysfunction in the PA-challenged group. The simultaneous accumulation of LC3-II and p62 (SQSTM1), accompanied by the appearance of swollen mitochondria and stalled autophagic vacuoles, suggested a severe accumulation of autophagosomes [14]. The bMECs initiated autophagosome formation but failed to complete lysosomal degradation, leading to toxic intracellular accumulation. Remarkably, SB treatment alleviated this accumulation, facilitating the clearance of p62 and the morphological recovery of mitochondria. This underscores that the protective efficacy of SB lies in its capacity to rescue the lysosomal degradation pathway, thereby preventing autophagic cell death [28]. The critical capacity of SB to modulate autophagic marker accumulation has also been increasingly documented in related epithelial stress models. Recent investigations have highlighted that SB ameliorates inflammatory barrier dysfunction and intracellular oxidative stress by promoting functional autophagy pathways [29,30]. Consistent with these findings, our observations indicate that in the context of bovine mastitis, SB not only dampens the initial inflammatory burst but critically intervenes to rescue lysosomal degradation. This comparative evidence further underscores the dual role of SB in maintaining both immune and intracellular metabolic homeostasis during severe bacterial challenges.

The concurrent regulation of MAPK and autophagy by SB points towards a sophisticated metabolic-immune crosstalk. Autophagic initiation and inflammatory resolution are intricately coupled processes governed by cellular energy sensors [31,32]. Future investigations should systematically explore the upstream metabolic sensors, particularly the AMP-activated protein kinase (AMPK) signaling axis, which acts as a master regulator of both autophagic flux and immune responses [33,34]. Understanding how the AMPK network—potentially alongside downstream transcriptional regulators like IRF1—drives the fundamental mechanisms of inflammatory resolution and orchestrates the phenotypic polarization of resident macrophages in the mammary microenvironment will further complete our mechanistic understanding of SB’s pharmacological targets [35].

Despite the promising findings, this study has limitations. The in vitro bMEC model, while robust for molecular dissection, cannot fully capture the complex cellular heterogeneity of the bovine udder, particularly the interactions between epithelial cells and resident immune cells. In addition, because pharmacological or genetic manipulation of TLR4/MAPK signaling (e.g., receptor antagonists, siRNA knockdown, or rescue experiments) was not performed, the present data establish a strong association rather than a proven causal role for the TLR4/MAPK axis in mediating the protective effect of SB; this causal relationship should be confirmed by future loss-/gain-of-function studies. Furthermore, given the emerging concept of the gut–mammary axis, the systemic bioavailability and immunomodulatory effects of exogenous SB supplementation via feed require extensive in vivo validation [36]. Additionally, the lack of an SB-alone group in our RNA-seq analysis limits the evaluation of SB’s baseline transcriptomic effects on uninfected cells.

## 5. Conclusions

Collectively, our study provides compelling evidence that sodium butyrate mitigates PA-induced apoptosis and inflammation, and these protective effects are closely associated with the suppression of the TLR4/MAPK signaling cascade and the alleviation of autophagic marker accumulation. These mechanistic insights not only broaden our understanding of PA pathogenesis in mastitis but also position SB as a non-antibiotic candidate for mitigating bovine udder infections.

## Data Availability

All transcriptomic data have been deposited in a public repository, https://ngdc.cncb.ac.cn/gsa/ (accessed on 3 September 2026), with GSA accession number: CRA048138. Other data are provided within the manuscript or as Supplementary Files.

## Supplementary Materials

The following supporting information can be downloaded at: https://www.mdpi.com/article/10.3390/vetsci13090925/s1, Table S1: Primers used for qPCR determination; Figure S1: Original images of Western-blotting.
