# Supplementary material for: Sodium Butyrate Mitigates Pseudomonas aeruginosa Infection in bMECs Associated with the Modulation of TLR4/MAPK Pathway and Improvement of Autophagic Markers
Source: Vet Sci. 2026 Sep 8;13(9):925. doi: 10.3390/vetsci13090925 (PMC13611944; doi:10.3390/vetsci13090925)
Supplement: Supplementary file 1 [file vetsci-13-00925-s001.zip › figure S1.pdf]

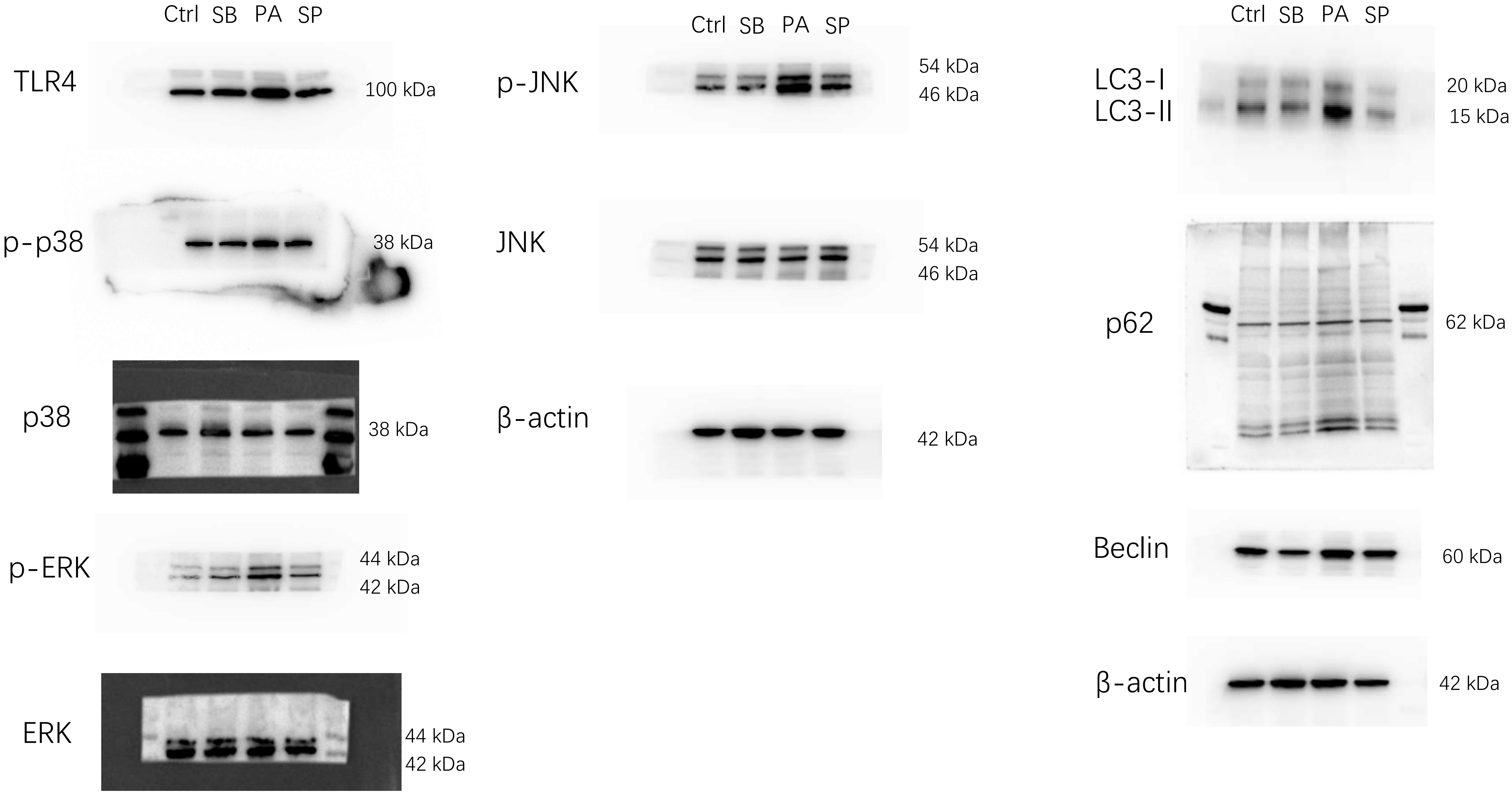

Ctrl SB PA SP
TLR4
100 kDa
p-p38
38 kDa
p38
38 kDa
p-ERK
44 kDa
42 kDa
ERK
44 kDa
42 kDa
Ctrl SB PA SP
p-JNK
54 kDa
46 kDa
JNK
54 kDa
46 kDa
β-actin
42 kDa
Ctrl SB PA SP
LC3-I
LC3-II
20 kDa
15 kDa
p62
62 kDa
Beclin
60 kDa
β-actin
42 kDa
