# Supplementary material for: Sodium Butyrate Mitigates Pseudomonas aeruginosa Infection in bMECs Associated with the Modulation of TLR4/MAPK Pathway and Improvement of Autophagic Markers
Source: Vet Sci. 2026 Sep 8;13(9):925. doi: 10.3390/vetsci13090925 (PMC13611944; doi:10.3390/vetsci13090925)
Supplement: Supplementary file 1 [file vetsci-13-00925-s001.zip › table S1.pdf]

Table S1 Primers used for qPCR determination.

Target genes	Primer sequence 5'-3'	Amplicon size
Tlr4	F:GCTGTTTGACCAGTCTGATTGC	102
	R:GGGCTGAAGTAACAACAAGAGGAA	
Mapk14	F:GGTCAGTGGGATGCATAATGG	101
	R:CCCCGTCAGACGCATAATCT	
Il6	F:CCAGAGAAAACCGAAGCTCTCAT	100
	R:CCTTGCTGCTTTCACACTCATC	
Il1b	F:ATTCTCTCCAGCCAACCTTCATT	100
	R:TTCTCGTCACTGTAGTAAGCCATCA	
Map1lc3b	F:CAAAATCCCGGTGATAATCGA	107
	R:TGACCAACTCGCTCATGTTGA	
Sqstm1	F: AAGGAGTGTCGGAGGGATCA	92
	R: CCGTTACAGCCGTCACAGAT	
Becn1	F:GCGATGGCAGTTTTGGAAGT	152
	R:CTGACACACGAAGCTCACCT	
Atg5	F:TAGCATCATCCCGCAACCAA	145
	R:GCTTGCAGCAGTGTTTCTGG	
Gapdh	F:TTGTCTCCTGCGACTTCAACA	103
	R:TCGTACCAGGAAATGAGCTTGAC	
Uxt	F:TGTGGCCCTTGGATATGGTT	101
	R:GGTTGTCGCTGAGCTCTGTG	
Rps9	F:CCTCGACCAAGAGCTGAAG	64
	R:CCTCCAGACCTCACGTTTGTTC	
